# Baboons (*Papio papio*) Process a Context-Free but Not a Context-Sensitive Grammar

**DOI:** 10.1038/s41598-020-64244-5

**Published:** 2020-04-30

**Authors:** Raphaëlle Malassis, Stanislas Dehaene, Joël Fagot

**Affiliations:** 1 Laboratoire de Psychologie Cognitive, Université d’Aix-Marseille, Marseille, France; 2 School of Psychology and Neuroscience, University of St Andrews, St Andrews, Fife, Scotland, United Kingdom; 3 Collège de France, Paris, France; 4 Cognitive Neuroimaging Unit, CEA DSV/I2BM, INSERM, Université Paris Sud, Université Paris-Saclay, NeuroSpin Center, 91191, Gif-sur-Yvette, France

**Keywords:** Psychology, Animal behaviour

## Abstract

Language processing involves the ability to master supra-regular grammars, that go beyond the level of complexity of regular grammars. This ability has been hypothesized to be a uniquely human capacity. Our study probed baboons’ capacity to learn two supra-regular grammars of different levels of complexity: a context-free grammar generating sequences following a mirror structure (e.g., AB | BA, ABC | CBA) and a context-sensitive grammar generating sequences following a repeat structure (e.g., AB | AB, ABC | ABC), the latter requiring greater computational power to be processed. Fourteen baboons were tested in a prediction task, requiring them to track a moving target on a touchscreen. In distinct experiments, sequences of target locations followed one of the above two grammars, with rare violations. Baboons showed slower response times when violations occurred in mirror sequences, but did not react to violations in repeat sequences, suggesting that they learned the context-free (mirror) but not the context-sensitive (repeat) grammar. By contrast, humans tested with the same task learned both grammars. These data suggest a difference in sensitivity in baboons between a context-free and a context-sensitive grammar.

## Introduction

**Figure 1:**
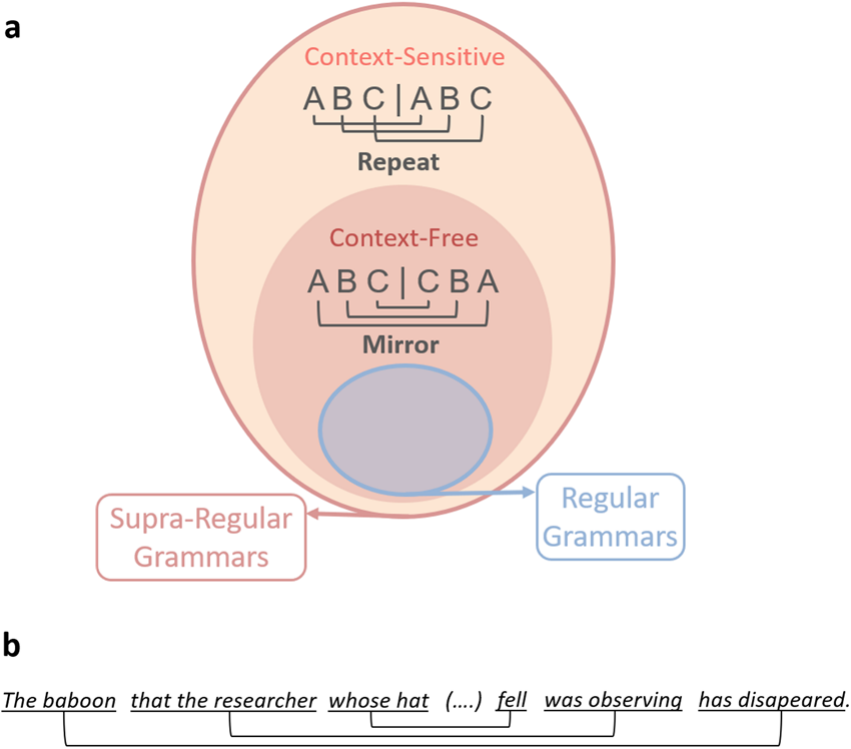
(**a**) Illustration of the organization of the regular and supra-regular classes of grammars within the Chomsky hierarchy. This hierarchy comprises a nested set of hierarchical classes (e.g., any system able to generate or produce supra-regular grammars can generate or produce regular grammars, whereas the contrary is not true). Within the broad class of supra-regular grammars, context-free grammars must be distinguished from context-sensitive grammars, the latter requiring greater computational power. The mirror grammar belongs to the class of context-sensitive grammars and involves centre-embedded dependencies. The repeat grammar belongs to the class of context-free grammars and involves crossed dependencies. (**b**) Centre-embedded dependencies between noun phrases and their corresponding verb phrases in English.

Assessing the syntactic abilities of various animal species is essential in order to better understand the evolution of the cognitive operations involved in human language processing^1–3^. This requires the use of a unified framework and efficient tools for quantifying syntactic complexity^2,4^. The most influential syntactic complexity metric is known as the “Chomsky hierarchy” (or the “formal language theory hierarchy”)^5^. It offers a typology of different categories of grammars of increasing level of complexity and describes the minimal computational devices required to generate and recognize them. Within this hierarchy, regular grammars are distinguished from more complex, supra-regular, grammars (Fig. 1a). Briefly, regular grammars can be generated by a finite-state automaton, solely based upon the labelling of transitions between a finite set of states; supra-regular grammars, however, can involve an arbitrary number of long-distance dependencies embedded within each other and require an additional memory device such as a stack^2,5^.

Humans have the ability to process sentences involving a variable number of remote dependencies. This is particularly well exemplified by our ability to understand and produce sentences in which noun phrases and their corresponding verb phrases can be embedded within other noun and verb phrases in English (Fig. 1b). Chomsky^5^ argues that the number of embeddings is unbounded: English grammar for instance allows for an unlimited number of nested dependencies (but see^6^). Because keeping track an unbounded number of remote dependencies is beyond the scope of finite-state automata (for a demonstration, see e.g^5,7^.), English and other natural languages grammars are classified as supra-regular grammars in Chomsky’s hierarchy.

It has been hypothesized that humans may be unique in their ability to process supra-regular grammars, other species being restricted to regular grammars (i.e. the “supra-regular distinctiveness hypothesis”)^2,8,9^. Over the past fifteen years, much effort has been devoted to assessing whether any other species can also master supra-regular grammars^8,10–16^. Although positive evidence has been obtained^10,12,13,16^, most of it remains controversial^11,17–19^.

Most previous comparative experiments focused on a single artificial grammar, usually denoted “A^n^B^n^”. This grammar generates sequences with any number *n* of elements from a category A, followed by the same number of elements from category B (e.g., AABB, AAABBB, etc.). To assess whether a sequence is grammatically correct, it is sufficient to count and compare the number of elements from each category and reject the sequence if those numbers are unequal. This counting strategy requires a supra-regular computational device^5^, because keeping track of an arbitrary (unbounded) number of A elements in order to match them with the number of B elements is something that a finite-state automaton cannot do^5,7^. Counting is however of little relevance regarding the cognitive operations involved in natural language processing^9^. Moreover, this grammar lends itself to the exploitation of alternative, low-level heuristics in non-human^11,15^ as well as human participants^6,20–22^. Two other supra-regular grammars have been proposed as better suited to assess other species’ ability to process supra-regular grammars, namely the “mirror” and “repeat” grammars^9,23^. These two grammars involve an unbounded number of long-distance dependencies, but belong to two different classes of supra-regular grammars. Pitting them against each-other therefore allows to situate more precisely a species’ grammar processing abilities within the Chomsky hierarchy.

The mirror grammar generates sequences in which the second half of each sequence mirrors the first half (i.e., the same items appear in reverse order), such as AB | BA, ABC | CBA. Mastering this grammar requires keeping track of an arbitrary number of centre-embedded long-distance dependencies (the first element must be matched with the last element, etc). This can be done only if the system possesses a formal equivalent of a push-down stack, allowing it to retrieve items in a “last-in, first-out” (LIFO) manner. The repeat (or “copy”) grammar generates sequences in which the second half of each sequence is an exact copy of the first half (e.g. AB | AB, ABC | ABC, etc.). Processing the repeat grammar requires keeping track of an arbitrary number of crossed dependencies, another type of long-distance relationship which requires a linear tape from which items can be retrieved in a “first-in, first-out” (FIFO) manner, starting with the first stored item.

In practise, possessing such a “tape” or FIFO stack does not seem more complicated than having a LIFO stack. Indeed, the capacity to repeat in forward order may be an elementary brain operation available to birds or rats^24–26^. Formally, however, in the framework of the Chomsky hierarchy, they differ: the mirror and repeat grammars are both supra-regular, but correspond to two different sublevels within this hierarchy, namely context-free and context-sensitive grammars (or “mildly context-sensitive” grammars”^7^) respectively (Fig. 1a), the later requiring greater computational power to be processed^5^. Where do natural languages lie within those sublevels? Centre-embedded constructions are found in many natural languages, including English (Fig. 1b), which therefore require at least context-free grammar to be generated. But crossed dependencies are also found in a small number of languages, such as Swiss German^27^, Dutch^28^ and Bambara^29^. Context-sensitive, or mildly context-sensitive grammars^30^ are therefore required to generate those languages. Moreover, under certain conditions human adults can process both types of grammars, whether these are implemented in sequences of linguistic (e.g. non-sense syllables^31,32^) or non-linguistic stimuli (e.g. visual shapes^33,34^). Overall these data indicate that processing context-free and non-context-free (i.e. at least mildly context-sensitive) supra-regular grammars lies within the scope of humans, and is not restricted to linguistic material.

Jiang and collaborators^16^ recently provided positive evidence for successful processing of the mirror grammar in a non-human primate species. In this study, two macaques were extensively trained to reproduce spatial sequences on a touchscreen that followed a mirror or repeat pattern. The macaques then successfully completed a comprehensive series of generalization tests that suggested a genuine grasp of the mirror grammar. Specifically, the macaques performed largely above chance with new sequences, including sequences of extended length and presented in novel configurations of locations on the screen. One caveat is in order, however. This study focused primarily on the mirror grammar, and the monkeys’ ability to generalize the repeat grammar was not assessed: during training, the macaques successfully learned to produce a set of length-4 (AB | AB) and length-6 (ABC | ABC) repeat sequences, but generalization of this behaviour to novel, untrained sequences was not tested.

As noted above, repetition of fixed behavioural sequences has also been reported in other species^24–26,35–37^, definitely showing that this ability is not restricted to our species. Whether such behaviour merely reflects the operation of a shallow, specialized neural repetition mechanism, however, or demonstrates a genuine grasp of the abstract properties of the repeat grammar, remains to be determined. A marker of a successful grasping of the repeat grammar lies in the ability to recognize it in novel sequences, including sequences of any length (as supra-regular grammars can involve an *arbitrary* number of long-distance dependencies). In other words, after having been trained to respond “1, 2” given “1, 2”, one should be able to respond “3, 4” given “3, 4”, as well as “3, 4, 5” given “3, 4, 5”. Additional generalization tests are therefore required to further assess non-human species’ ability to process supra-regular grammars of a higher level of complexity than context-free grammars.

In this context, the purpose of the present experiments was threefold: (1) to replicate, in another old-world monkey species, the Guinea baboon (*Papio papio*), Jiang *et al*.’s finding that the mirror grammar can be learned by non-human primates; (2) to determine whether baboons could learn and generalize the repeat grammar; and (3) to assess those questions using a prediction task, in which the animals perform a simple target-tracking task, and learning is solely evidenced by a response-time benefit^38–40^.

## Results

**Figure 2:**
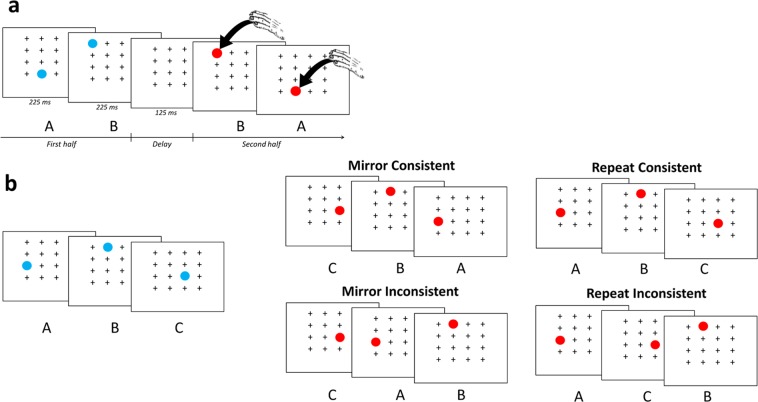
(**a**) Illustration of the general principle of the task. On a given trial, the first half of a target sequence is displayed on a grid of 16 possible locations on a touchscreen. After a 125 ms delay, the second half of the sequence is displayed in the same matrix and the baboon now has to touch each target as soon as it appears. In this example, a length-4 training sequence following the mirror grammar (AB | BA) is presented. (**b**) Structures of the length-6 sequences presented in Tests 1–3 and control: example of a sequence first half (left panel) and the corresponding second half for each experimental condition (right panel).

Fourteen Guinea baboons (*Papio papio*) were tested using an adaptation of the serial response time task^38–40^. Each trial followed the general procedure illustrated in Fig. 2a. The first half of a target sequence was sequentially displayed on a grid of 16 possible locations on a touchscreen. Each target (a blue dot) appeared for 225 ms, and then appeared at the next location for the same duration, with no delay. No response was required from the participant at this stage. After a delay of 125 ms, the second half of the target sequence (either a mirror or a repeat of the previous sequence, depending on the experiment) was sequentially displayed in the same matrix and the participant now had to touch each target (a red dot) as soon as it appeared. The accuracy of the touch as well as the response time (RTs) were recorded for every target. Serial response time tasks of this kind often induce almost no error^41^, and that was also true in the current research with baboons (Mean correct ± SD = 96.22 ± 01.02%). However, variation in response time provides critical information on participants’ expectations about the locations of the incoming targets, i.e. their sensitivity to the grammar underlying the target displacements. Here, our logic was to present sequences in blocks in which the second half of the sequence was predictable from the first half (being either a repetition or a mirror of the first half). If baboons learned this rule, even implicitly, then we would expect their response times to become faster on such predictable trials, compared to trials in which the rule is violated.

The baboons received two consecutive experiments in a within-participants design, which were presented in a counterbalanced order across participants. One of these experiments tested the learning and generalization of the mirror grammar. The second tested the learning and generalization of the repeat grammar. Each experiment began with an initial exposure phase comprising 40 training blocks of 96 trials using a set of 64 length-4 sequences that all followed the intended grammar (mirror or repeat). Sequences were of the form AB | BA in the mirror experiment, and AB | AB in the repeat experiment. Baboons’ understanding of the grammars was then assessed using a series of three tests relying on a violation procedure. The rationale behind this procedure was that if baboons are sensitive to the grammars underlying the target’s displacement, violations of their expectations should result in slower RTs. In Test 1, baboons’ responses to the following two types of length-6 test sequences were compared: novel consistent sequences that followed the intended grammar, and novel inconsistent sequences that involved a violation of the grammar (Fig. 2b). Consistent sequences were of the form ABC | CBA in the mirror experiment, and ABC | ABC in the repeat experiment. In inconsistent sequences, the last two targets were presented in a reversed order (mirror experiment: ABC | C**AB**, repeat experiment: ABC | A**CB**; violations in bold). The sequences were matched between conditions to prevent learning of the test sequences and control motor constraints (see Methods section for a full description of the procedures used to construct the sequences).

Previous research has suggested that participants may rely on the recognition of fragments of training sequences within novel test sequences, instead of processing the underlying grammar^17,18^. To exclude this possibility in the current study, we manipulated in Tests 2–3 the familiarity of the length-4 subsequences contained in the length-6 test sequences. In Test 2, the length-6 test sequences were all constructed from length-4 training sequences (e.g. training mirror sequences 43 | 34 and 53 | 35 were used to create the mirror consistent sequences 543 | 345 and 453 | 354, and the mirror inconsistent sequences 453 | 345 and 543 | 354; numbers denote locations on the touchscreen, see Supplementary Fig. 1; see also Methods section for further details). On the contrary, neither the consistent nor the inconsistent sequences from Test 3 contained any length-4 training sequence (see Supplementary Table 2 for a full list of the sequences used in the different phases). Therefore, if baboons relied on rote memorization of the training sequences, they should succeed in Test 2 but fail to notice the violations occurring in Test 3.

**Figure 3:**
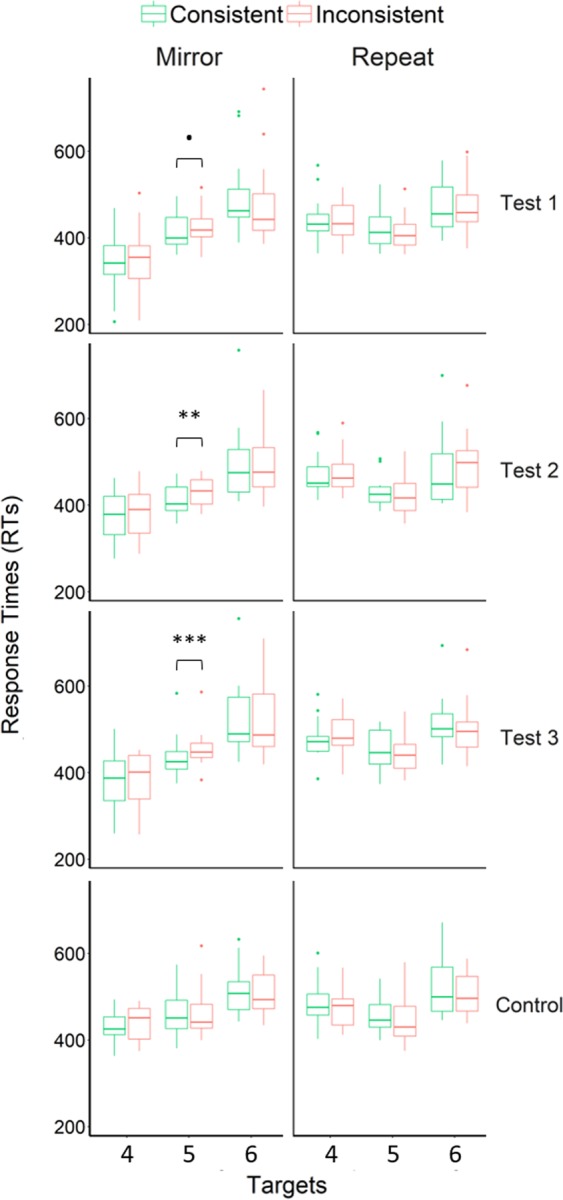
Baboon results. Boxplots of participants’ mean RTs, for each experiment and test. Dots represent outliers. Asterisks indicate significant differences in RTs between consistent and inconsistent sequences (see main text), .p < 0.10; **p < 0.01, ***p < 0.001.

A test was considered as successfully passed if RTs were significantly slower in the inconsistent compared to the consistent condition. A repeated-measures ANOVA was conducted on RTs, involving the condition (Consistent, Inconsistent), experiment (Mirror, Repeat), test (1–3), and target number (4–6) as within-participant factors (Fig. 3, see also Table 1 in Supplementary Information for a summary of mean RTs and standard deviations in each testing condition). We found a significant interaction between condition, experiment and target number (F (2, 26) = 7.43, *p* = 0.003, η_p_^2^ = 0.37). Pairwise comparisons revealed slower RTs on the first violation (Target 5) occurring in inconsistent mirror sequences (Mean ± SD = 439 ± 36 ms) compared to consistent sequences (420 ± 36 ms), t (13) = 5.29, *p* < 0.001, Bonferroni-Holm adjusted alpha level = 0.05/(6 − 1 + 1) = 0.008, Cohen’s *d* = 0.53 (see Data analyses section regarding how Bonferroni-Holm adjusted alpha level were calculated). All remaining comparisons were non-significant (*p*s > 0.05). This suggests that baboons successfully processed the overall structure of the mirror sequences, as shown by their reaction to the first violation of that structure occurring in inconsistent sequences. By contrast, baboons’ RTs were not slower in response to the violations occurring in repeat sequences (consistent sequences: 435 ± 39 ms, inconsistent sequences: 426 ± 42 ms on Target 5), suggesting that they did not process the repeat grammar in the current task. The four-way interaction involving the condition, experiment, test, and target number as factors was not significant (*p* = 0.44). An ANOVA conducted on RTs obtained on Target 5 moreover indicated a significant interaction between experiment and condition, F (1, 13) = 26.10, *p* < 0.001, η_p_^2^ = 0.67, confirming that the baboons behaved differently in response to violations in the mirror and repeat experiments.

**Table 1:** Overview of the experimental timeline and summarized descriptions of the different phases.

Phases	Description	Conditions	No. of trials by block	No. of blocks	Sequences structure		
					Mirror experiment	Repeat experiment	
**Exposure**	Length-4 training sequences	Baseline	96	40	AB|BA	AB|AB	
**Test 1**	Length-6 sequences	Baseline Consistent Inconsistent	60 20 20	4	ABC|CBA ABC|CBA ABC|CAB	ABC|ABC ABC|ABC ABC|ACB	
Interruption (2 months)							
**Control**	Presentation of the different conditions at equal frequencies	Mirror-Consistent Mirror-Inconsistent Repeat-Consistent Repeat-Inconsistent	16 16 16 16	4	ABC|CBA ABC|CAB ABC|ABC ABC|ACB		
**Exposure**	Length-4 training sequences	Baseline	96	20	AB|BA		AB|AB
**Test 2**	Length-6 sequences containing length-4 training sequences	Consistent Inconsistent Baseline Consistent Inconsistent	16 16 48 16 16	4	ABC|CBA ABC|CAB ABC|CBA ABC|CBA ABC|CAB		ABC|ABC ABC|ACB ABC|ABC ABC|ABC ABC|ACB
**Test 3**	Length-6 sequences made of novel targets combinations						

Additional post-hoc comparisons revealed that baboons were slower to respond to the first violation occurring in inconsistent mirror sequences in all tests, but that this difference reached significance for Tests 2 and 3 only (Test 1: t (13) = 2.03, *p* = 0.06, Bonferroni-Holm adjusted alpha level = 0.05/(3 − 3 + 1) = 0.05 = 0.05, Cohens’ *d* = 0.33; Test 2: t (13) = 3.83, *p* = 0.002, Bonferroni-Holm adjusted alpha level = 0.05/(3 − 2 + 1) = 0.025, Cohens’ *d* = 0.66; Test 3: t (13) = 4.80, *p* < 0.001, Bonferroni-Holm adjusted alpha level = 0.05/(3 − 1 + 1) = 0.017, Cohens’ *d* = 0.42). Importantly, these post-hoc comparisons confirmed that baboons reacted to violations of the mirror grammar occurring in test sequences containing familiar training sequences (Test 2), as well as to those occurring in sequences made of entirely novel combinations of locations (Test 3). Consequently, baboons’ results in the mirror experiment cannot be accounted for by rote learning of the training sequences.

In addition to this difference in RTs on Target 5 between consistent and inconsistent mirror sequences, a visual inspection of Fig. 3 revealed differences in responses speed across targets. A recency effect, corresponding to faster RTs on the targets seen the most recently, was notably observed in the mirror experiment: baboons were especially fast to respond to Target 4 (Mean ± SD = 367 ± 66 ms), slower to respond to Target 5 (430 ± 35 ms, t (13) = 4.40, *p* < 0.001, Bonferroni-Holm adjusted alpha level = 0.05/2 − 1 + 1 = 0.25, Cohens’ *d* = 1.18), and slower again to respond to Target 6 (502 ± 80 ms, t (13) = 4.33, *p* < 0.001, Bonferroni-Holm adjusted alpha level = 0.05/2 − 2 + 1 = 0.05, Cohens’ *d* = 1.17). These data are consistent with previous reports of recency effects in sequence learning experiments in monkeys using a short delay between sequence presentation and test^42^. Moreover, similar data indicating a memory decay with ordinal position were observed in macaques who successfully produced mirror sequences in Jiang *et al*.^16^ study. However, one important question arising from this observation is whether this recency effect could explain the difference in RTs observed between consistent and inconsistent mirror sequences. One structural property of our sequences is that Target 5 has been seen more recently in consistent mirror sequences (A**B**C | C**B**A) than in inconsistent sequences (**A**BC | C**A**B). The slower RTs observed on Target 5 in inconsistent sequences could therefore result from differences in sequences structures across conditions, rather than detection of violations of the mirror grammar. The fact that the difference in RTs did not reach significance for Test 1, and that the lowest effect size was observed for this test, suggests that the baboons benefited from the additional 20 exposure blocks they have performed between Test 1 and Tests 2–3 (see Methods section for a detailed timeline of the experiments). These data are consistent with the hypothesis that monkeys need extended exposure to the mirror grammar to learn it^16^. Alternatively, greater performance in Tests 2-3 may results from exposure to length-6 mirror sequences provided during Test 1. In other words, baboons may have been familiarized with length-6 mirror sequences during that test, leading to better performance in subsequent tests. To test this hypothesis, we assessed whether baboons’ performance improved in the course of Test 1. We compared the difference in RTs on Target 5 between consistent and inconsistent mirror sequences, in the first half of Test 1 (Blocks 1-2, i.e. 40 trials per monkey and condition) and its second half (Blocks 3-4). No significant difference was found (Blocks 1-2: Mean ± SD of the difference = 13 ± 40 ms, Blocks 3-4: 16 ± 22 ms, t (13) = 0.22, *p* = 0.83). These data therefore do not support the hypothesis that baboons beneficiated from exposure to mirror sequences of extended length during Test 1, but beneficiated instead from the 20 additional training blocks provided between Test 1 and Tests 2-3. Altogether, these results suggest that baboons demonstrated learning of the mirror grammar, as evidenced by an increase in RTs on the first violation occurring in inconsistent sequences, after ~4000 training trials.

To assert whether the above results indicate a genuine processing of the mirror grammar – one that cannot be explained by differences in sequence structure across conditions, independently of any learning – we exposed the baboons to a control that used the same sequences as in Test 3 but presented them at equal frequencies (i.e., 25% of the trials were mirror consistent sequences, 25% mirror inconsistent, 25% repeat consistent, 25% repeat inconsistent, randomly intermixed). If a mere recency effect can explain the difference in RTs observed between consistent and inconsistent mirror sequences, we should observe a similar pattern of results in the control as in Tests 1-3. This control was presented between Test 1 and Tests 2-3 after a two-month break in the experiment. Unlike the main experiment, no difference in RTs on Target 5 was observed between the four conditions, F (3, 39) = 0.77, *p* = 0.52 (Fig. 3). These results therefore confirm that the increase in RTs on the first violation occurring in inconsistent mirror sequences observed in Tests 1–3 does not result from recency effects.

**Figure 4:**
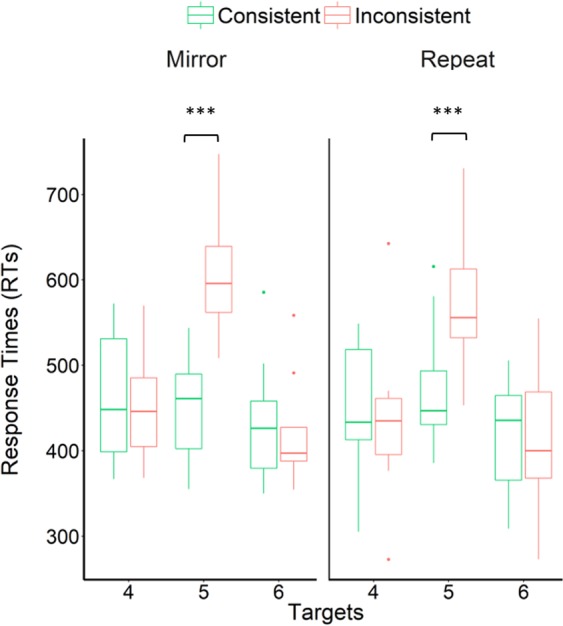
Human results. Boxplots of participants’ mean RTs, for each experiment. Dots represent outliers. Asterisks indicate significant differences in RTs between consistent and inconsistent sequences (see main text), ***p < 0.001.

Finally, human adults were tested using the same task. Eight participants performed the mirror and repeat experiments in a within-participants design (presented in a counterbalanced order across participants). The human experiments settings were overall the same as in the baboon experiments (see *Methods* section), except that the experiments length was reduced. After exposure to the mirror or repeat grammar in length-4 sequences during two 64-trial blocks, the participants were presented with Test 3. A repeated-measures ANOVA was conducted on RTs, involving the condition (Consistent, Inconsistent), experiment (Mirror, Repeat), and target number (4–6) as within-participant factors (Fig. 4). We found a significant interaction between condition and target number (F (2, 14) = 14.23, *p* < 0.001, η_p_^2^ = 0.67). Pairwise comparisons revealed overall slower RTs on the first violation (Target 5) occurring in inconsistent sequences (Mean ± SD = 593 ± 71 ms) compared to consistent sequences (461 ± 64 ms), t (7) = 4.70, *p* = 0.002, Bonferroni-Holm adjusted alpha level = 0.05/(6 − 2 + 1) = 0.016, Cohen’s *d* = 1.93. All remaining comparisons were non-significant (*p*s > 0.05). No interaction between condition, target and experiment was found (*p* > 0.05). Humans therefore showed a selective increase in RTs on the first target on which the violation occurred in mirror (Mean ± SD on Target 5 = 607 ± 74 ms for inconsistent sequences, 450 ± 70 ms for consistent sequences; t (7) = 4.79, *p* = 0.002, Bonferroni-Holm adjusted alpha level = 0.05/(2 − 1 + 1) = 0.025, Cohen’s *d* = 2.17) as well as repeat sequences (578 ± 90 ms for inconsistent sequences, 472 ± 83 ms for consistent sequences; t (7) = 3.18, *p* = 0.02, Bonferroni-Holm adjusted alpha level = 0.05/(2 − 2 + 1) = 0.05, Cohen’s *d* = 1.23), indicating that they successfully learned both grammars in the current task. A repeated-measures ANOVA performed on RTs obtained on Target 5 moreover revealed no interaction between experiment and condition (*p* = 0.22): contrarily to baboons, humans have not responded differently to the two grammars. Participants were in addition asked at the end of each experiment to verbally describe the rule which according to them was followed by the sequences: all of them successfully reported that the sequences had a mirror or repeat structure.

Other uniquely human aspects are the absence of a recency effect and a much larger size of the inconsistency effect (157 ms in the mirror experiment in humans, 19 ms in baboons). These conclusions were backed up by an ANOVA where the species (Baboons, Humans) was introduced as an additional factor, performed on data from Test 3 of the mirror experiment. We found a significant interaction between the species, target and condition, F (2, 40) = 16.98, *p* < 0.001, η_p_^2^ = 0.46. Post-hoc tests assessing differences in RTs between baboons and humans for each target and condition confirmed that baboons tended to be faster to respond to Target 4 compared to humans, both for consistent (382 ± 70 and 463 ± 74 ms, respectively, t (20) = 2.48, *p* = 0.022, Bonferroni-Holm adjusted alpha level = 0.05/(6 − 3 + 1) = 0.013, Cohen’s *d* = 2.17) and inconsistent sequences (385 ± 65 and 452 ± 65 ms, respectively, t (20) = 2.34, *p* = 0.030, Bonferroni-Holm adjusted alpha level = 0.05/(6 − 5 + 1) = 0.025, Cohen’s *d* = 2.17), and tended to be slower than humans on Target 6 (consistent condition: 525 ± 87 and 440 ± 78 ms, respectively, t (20) = 2.39, *p* = 0.027, Bonferroni-Holm adjusted alpha level = 0.05/(6 − 3 + 1) = 0.017, Cohen’s *d* = 1.03; inconsistent condition: 521 ± 92 and 426 ± 69 ms, respectively, t (20) =2.65, *p* = 0.015, Bonferroni-Holm adjusted alpha level = 0.05/(6 − 2 + 1) = 0.010, Cohen’s *d* = 1.17). On Target 5, baboons and humans responded at similar speed to consistent sequences (437 ± 50 and 459 ± 67 ms, respectively, t (20) = 0.51, *p* = 0.61, Bonferroni-Holm adjusted alpha level = 0.05/(6 − 6 + 1) = 0.05), while humans were much slower to respond to inconsistent sequences compared to baboons (607 ± 74 and 457 ± 46 ms, respectively, t (20) = 5.91, *p* < 0.001, Bonferroni-Holm adjusted alpha level = 0.05/(6 − 1 + 1) = 0.008).

## Discussion

Our study compared a non-human primate species’ sensitivity to a context-free (mirror) and a context-sensitive (repeat) grammar. In Tests 1–3, baboons failed to react to violations occurring in inconsistent repeat sequences, but they repeatedly reacted to violations introduced within the mirror sequences. Test 3 excluded the possibility that baboons relied on the recognition of fragments of the previously trained sequences, and a control confirmed that the pattern of results observed in Tests 1–3 was a genuine consequence of learning. These data therefore suggest that the baboons learned, to some extent at least, the context-free mirror grammar in the current task, while we found no evidence that they learned the context-sensitive repeat grammar. Computationally, at least according to formal language theory, the context-sensitive grammar we tested is more complex since it requires, at a minimum, a first-in-first-out stack, and therefore the ability to store all sequences items and then return to sequence onset. By contrast, the context-free mirror grammar is computationally simpler as it requires only a push-down stack with a last-in-first-out mechanism. Our results suggest that baboons have access to the latter but not the former, at least under the current learning conditions.

Comparison between baboons’ and humans’ performance in this task moreover revealed some similarities as well as differences between the two species. Results obtained in humans converge with those of baboons in two respects. First, the two species showed a selective increase in RTs on the first violation occurring in mirror sequences (Target 5). Second, neither humans nor baboons showed a slowdown on violations occurring on the following target (Target 6) in those sequences. This can tentatively be accounted for by the fact that the last target location can be predicted in both the consistent and inconsistent conditions, as it is the only location from the first half of the sequence that has not been presented yet. These results suggest that both species successfully learned the mirror grammar in the current task.

However, four important differences were also observed between the two species. Firstly, humans reacted to violations of both the mirror and repeat grammars, contrarily to baboons. These data, showing that humans computational abilities were not restricted to the context-free grammar in the current task, are consistent with previous reports from the literature^31–34^. Secondly, humans’ RTs remained flat across targets. Baboons’ RTs, instead, were dominated by a recency effect in the mirror experiment, as indicated by increasing RTs as a function of ordinal position. Our control experiment ruled out the possibility that baboons’ increase in RTs on violation occurring in mirror sequences in Tests 1-3 could be explained by this recency effect, but it nevertheless warrants discussion. Similar data suggesting a memory decay with ordinal position (indicated by both increasing RTs and decreasing accuracy) were reported by Jiang *et al*.^16^ when macaques produced mirror sequences. The fact that no recency effect was observed in humans may result from differences in working memory span between human and non-human primates. A study comparing humans’ and baboons’ span in an immediate serial spatial recall task^35^ reported almost no memory decay in humans for sequences of three targets, while important variations in accuracy as a function of ordinal position were observed for sequences of longer length. By contrast, baboons showed variations in accuracy across ordinal positions even for short sequences of three locations. Further experiments will have to be conducted in humans to assess whether humans would show variations in RTs across ordinal positions similar to those observed in baboons and macaques, when presented with mirror sequences of longer lengths. Note also that we tested students and that a different picture might be obtained in other human populations, as memory span is known to be affected by factors such as age and education^43–45^. Although recency effects do not account for RTs differences between baseline and violation trials, they might partly explain why learning was limited to mirror sequences in baboons in the current study. We speculate that recency effects may have promoted last-in-first-out strategies required to encode mirror sequences, because the last item of the first half of the sequence, characterized by a stronger memory trace, is precisely the first item that must be recalled in mirror sequences. By contrast, recency effects may hamper learning of the repeat grammar in the current task, because the baboon must refrain its response to this last item to adopt a first-in-first-out strategy. This effect was absent in our group of human participants who had no clear recency effect in the current study and learned the two grammars. Thirdly, important differences in learning speed were observed between the species: Despite much longer exposure provided to baboons compared to humans (~100 trials), baboons showed their best performance in Test 2-3, i.e. after ~4000 training trials, while the effect of sequences inconsistency was only marginally significant for Test 1 (i.e. after ~2000 training trials). These data converge with those of Jiang *et al*.^16^, in which macaques only exhibited the capacity to produce mirror sequences after tens of thousands of training trials, whereas human pre-schoolers required only about five trials. Finally, a much larger size of the inconsistency effect was observed for humans compared to baboons.

These discrepancies in learning speed and inconsistency effect size may reflect the involvement of different types of learning processes in the two species. All human participants accurately reported that the sequences followed a mirror or a repeat rule, indicating that they acquired explicit knowledge about the sequences’ structure. In contrast, baboons may have relied solely on implicit learning processes. One possibility is that only humans have access to an explicit learning algorithm that relies on the selection among a set of discrete, symbolic hypotheses about the grammars underlying the data – a hypothesis that has been independently proposed by several groups (e.g.^46–48^). Such an algorithm would allow for a much faster acquisition speed, whereas generic algorithms for implicit learning based on neural networks such as Long Short-Term Memory (LSTM), while also capable of learning some context-free grammars, do so at a very slow pace, perhaps comparable to baboons (see e.g.^49^). Little attention has been devoted to the study of implicit *versus* explicit learning of sequential regularities in non-human species so far and future research will need to address this issue.

In summary, this study suggests a difference in sensitivity in baboons between a context-free (mirror) and a context-sensitive (or middle-context-sensitive, repeat) grammar, as we found evidence of learning only for the former in the current task. Moreover, several differences were observed between the pattern of responses obtained in baboons and humans tested with the same task, including the fact that baboons’ RTs were affected by a strong recency effect, and only modestly affected by the consistency of the sequences. Thus, even if baboons learned the mirror grammar in the current task, this regularity was not the major determinant of their responses, contrary to humans. Baboons moreover needed much more exposure compared to humans to learn this grammar.

We close by drawing attention to some of the limits of the task used in the current study. First, we used a prediction task, where the participants merely *responded* to sequences of spatial targets and learning was assessed by a response time benefit when the sequences respected the grammar. These results imply that the baboons’ grasp of the context-free mirror grammar was sufficiently automatic as to occur under such experimental conditions, after ~4000 training trials. However, we cannot exclude that they would also have succeeded with the repeat grammar if the sequence-learning task had been more explicit, such as the production task used in Jiang *et al*.^16^, or involved training to perform repeat sequences of extended length. Secondly and relatedly, unlike more classical operant conditioning experiments, baboons were not differentially rewarded as a function of their performance in the current task (i.e. they received a food reward at the end of each trial, regardless of whether or not they learned the regularities underlying the targets displacements). This implies that baboons were rewarded during test trials, and could perhaps use this reinforcement as a basis for further learning. Note, however, that baboons were rewarded equally for responding to consistent and inconsistent sequences, and our analyses did not provide evidence that they learned to respond to the sequences of extended length during Test 1. Nevertheless, we cannot definitely rule-out the hypothesis that baboons were familiarized to some extent to length-6 sequences during Test 1. Thirdly, the participants were not required to perform the whole sequences but had to respond to the second sequence half after passively viewing its first half. On the one hand, this means that the monkeys could not have learned a mere “move forward, move backward” motor routine, as distinct components of visuospatial memory and motor planning were involved in our task. On the other hand, this feature of our design does not allow to determine how baboons would perform in a task requiring them to respond to whole mirror sequences. Finally, like Jiang *et al*.^16^ we used a visuo-motor task, and it remains unknown whether monkeys’ learning capacities are limited to this domain for supra-regular grammars. Further research is needed to assess whether similar results could be obtained for instance in a prediction task involving auditory input (for an example of such paradigm that could be adapted to non-human primates, see e.g.^41^). This question is particularly important in two respects. First, it would allow to study whether supra-regular computational capabilities are underlined by domain- and modality-specific learning mechanisms, or by a more domain-general ability. In humans, it could ultimately help to uncover whether the supra-regular computational capacities measured in such visuo-motor tasks are the same as those used in language. Secondly, it would shed light on the evolutionary functional origins of supra-regular computational capabilities. If it turns out that successful processing of this type of grammar cannot be found in other domains/modalities in non-human species, this could suggest that these capacities originated from the visuo-motor domain, and were later exploited in other domains during recent human evolution^50^.

To conclude, following Jiang *et al*.’s^16^ recent findings on macaques, the current study suggests that supra-regular computational capacities are within the grasp of baboons, another old world monkey species. This convergence supports the hypothesis that such a capacity was present in our last common ancestor with these species (25–30 million years ago)^51–54^. Human singularity may still lies in an unusual propensity to learn supra-regular structures^2,50^, an idea which is supported by the major difference in learning speed observed between humans and monkeys. In addition, it could be that the human/non-human limit involves higher levels of grammatical complexity, non-human primates’ syntactic abilities being possibly restricted to context-free grammars, or even to a subset of them. The present work tested only a single context-free grammar (mirror), which offers no guarantee that baboons would learn others as well. It is however worth noting that using a different task, Jiang *et al*.^16^ found that macaque monkeys could produce both mirror and repeat sequences. Although generalization of the repeat (i.e. context-sensitive, or middle-context-sensitive) grammar was not assessed in Jiang *et al*.’s study, these positive results call for further investigation. Rather than focusing on the broad distinction between regular and supra-regular grammars, future studies may therefore need to take a fine-grained approach to investigate where different species’ syntactic abilities precisely fall within the sublevels of both regular^55^ and supra-regular grammars^9^. Finally, human languages do not involve centre-embedded dependencies between identical elements, such as in the mirror grammar, but between functional categories (e.g. between different noun phrases and their corresponding verb phrases, as illustrated in Fig. 1b). Processing this kind of centre-embedded structures involve other cognitive abilities in addition to the ability to process context-free grammars, including the ability to learn dependencies between functional categories, which may not be in the scope of other species^56^.

## Methods

### Participants and apparatus

#### Baboons

Participants were 14 Guinea baboons (*Papio papio*, 8 females, age range 4-21 years) from the CNRS primate facility (Rousset-sur-Arc, France). Baboons lived within a larger group of 26 individuals, in a 700 m^2^ outdoor enclosure with access to indoor housing, and had a permanent access to ten Automated Learning Devices for Monkeys (ALDM; for a detailed description, see^57,58^) equipped with a 19-inch touch screen and a food dispenser. A key feature of ALDM is a radio frequency identification (RFID) reader that can identify individual baboons through microchips implanted in their arm. The baboons therefore participate in the research at will, without having to be captured, as the test programs can recognize them automatically. This research was carried out in accordance with French standards and received ethical approval from the French Ministry of Education (approval APAFIS#2717-2015111708173794 v3). The experiment was controlled using EPrime software (Version 2.0, Psychology Software Tools, Pittsburgh).

#### Humans

Eight university students participated in this study (five females, age range 21-28). All participants were paid for their participation, had normal or corrected-to-normal vision, and were naïve as to the goal of the study. Testing lasted approximately 30 minutes per participant and was divided in two sessions (one for each experiment) separated by a 5 minutes break. Written informed consent was obtained from all participants.

### Procedure

#### Task

The general procedure of the task is illustrated in Fig. 2a. Each trial began with the display of a fixation cross (120 × 120 pixels) presented at the bottom of the screen. Touching this stimulus triggered the display of 15 white crosses (60 × 60 pixels each) in addition to one target stimulus (a blue circle of 80 × 80 pixels) which were all displayed on a 16 (4 ×4) matrix. This first display was presented for 225 ms, and then the target moved with no delay to a different location for another period of 225 ms (its previous location being replaced by a cross). This procedure was repeated for either two or three different targets, depending on the sequence length. Following this, the computer presented a 125 ms display devoid of targets to signal the middle of the sequence. Then, a red instead of a blue target was used in the second half of the sequence to facilitate the distinction between the first and second halves. At this stage, baboons had to touch the red target once it appeared on the screen, and the accuracy of the touch and the corresponding response time were recorded for each target. A correct touch of the target immediately triggered the reappearance of the target at another location of the matrix, and this procedure was repeated until the end of the sequence. Trial ended when the participant touched the last target of the sequence. An accurate completion of the trial delivered grains of dry wheat inside the ALDM test unit. An incorrect response (i.e., selection of an incorrect location, or a touch on the screen during the first stage of the trial or during the interval signaling the middle of the sequence) stopped the trial and triggered a 5 s timeout without food reward. Trials in which the participants failed to select a stimulus within 5 s were aborted and presented again in the next trial. This procedure was followed for sequences of four (exposure phase) and six (test phases) targets, with the exception that the interval signaling the middle of the sequence lasted 300 ms instead of 125 ms in the test phases.

#### Experimental timeline

Table 1 describes the experimental timeline and provides detailed information on the training and test blocks as well as the sequence structures. The timeline was identical for mirror and repeat experiments, and the baboons were sequentially tested in these two experiments, with the experiment order being counterbalanced across participants. The baboons were first exposed to 40 blocks of 4-target sequences following a mirror or repeat structures. They were then submitted to Test 1 and the control test. To limit the potential influence of prior exposure to the grammar, the control test was administered after a two-month break during which the subjects were exposed to unrelated cognitive tasks. Twenty blocks of exposure trials were then given to the baboons, before submitting them to Tests 2 and 3, which were conducted concurrently (the different trial types involved in these tests being randomly intermixed). After completing all the phases of their first experiment (i.e., repeat or mirror experiment), the baboons participated in unrelated tasks for a further period of two months before being presented with the second experiment.

#### Block construction

The exposure phases used 64 length-4 training sequences. Tests 1–3 used three types of length-6 sequences, randomly intermixed: baseline sequences (of the form ABC | CBA in the mirror experiment and ABC | ABC in the repeat experiment), consistent sequences (ABC | CBA and ABC | ABC, respectively) and inconsistent sequences (ABC | CAB and ABC | ACB, respectively; see Table 1 for the exact numbers of each sequence type presented within a block for each test). Consistent and inconsistent sequences were presented at equal frequencies, and the sequences generated for each condition were matched in order to prevent learning of the test sequences during testing as well as differences in motor constraints across conditions (see the Sequence construction section below for more details). The length-6 baseline sequences were presented during one 96-trial trial block before each test, to familiarize the baboons with the display of three targets (instead of two in the exposure phases) within the first stage of the trials. In the control phase, mirror consistent, mirror inconsistent, repeat consistent, and repeat inconsistent sequences were presented at equal frequencies (1/4 of trials each), randomly intermixed. One 96-trial block of familiarization to length-6 sequences was administrated before the control as well, having the same balanced structure (1/4 of each sequence type).

#### Sequence construction

Unless otherwise noted in the subsections below, for each phase the sequences were generated with the following constraints: (1) each location on the touchscreen was presented within each serial position (Target 1, Target 2, etc) with equal frequency; (2) repetitions of the same target location were avoided within a sequence half; (3) the first halves of the sequences were identical in the consistent and inconsistent conditions, rendering the second half of the sequences unpredictable on that basis (i.e. no learning of the novel sequences could take place throughout testing); and (4) the second halves of the sequences were identical in the mirror consistent, mirror inconsistent, repeat consistent and repeat inconsistent conditions, thereby controlling for motor constraints across conditions. Sequences from the different conditions therefore differed from each other only regarding the matching between the two halves of the sequences. Custom-written Python code (www.python.org) was used to generate the sequences. The list of all the sequences used in each phase is available in Supplementary Table 2. Further details concerning the sequence construction procedure used are provided below for each phase:

**Exposure**. Sixty-four length-4 training sequences were generated. Each of the 16 locations of the matrix was presented four times in the first position of the sequence and combined with four different locations presented in second position of the sequence (i.e. 16 × 4 = 64 sequences; transitional probabilities between the first and second locations = 0.25).

**Test 1**. We randomly split the 16 possible locations into two sets. A first set of six locations (locations 1, 2, 3, 10, 13 and 15) was used to generate six baseline sequences. The remaining 10 locations were used to generate 80 test sequences (20 sequences per condition and experiment). Consistent and inconsistent sequences of Test 1 were therefore made of target locations which had never occurred in length-6 sequences before.

**Tests 2-3**. A set of 48 length-6 baseline sequences was generated for Tests 2–3, using the 16 locations of the matrix. Thirty-two (16 consistent and 16 inconsistent for each experiment) test sequences were generated for Test 2 from combinations of length-4 training sequences. The following procedure was used to generate the sequences used in Test 2: Two training sequences (e.g., 43 | 34 and 53 | 35) were recombined in order to create two consistent sequences that included them as their inner subsequences (543 | 345 and 453 | 354). Inconsistent sequences were constructed using the same training sequences in such a way that the two halves of the subsequence did not match between each other (e.g., 453 | 345 and 543 | 354). The same number of sequences was generated for Test 3 (16 consistent and 16 inconsistent per experiment), using combinations of locations which were not presented in any of the length-4 training sequences. Considering the limited number of possible sequences within the matrix of 16 locations, it was not possible to control for the frequency of each location within each serial position while also contolling for the familiarity *versus* novelty of the combinations of locations. The former variable was therefore not controlled during that phase.

**Control**. The same set of mirror consistent, mirror inconsistent, repeat consistent and repeat inconsistent sequences as in Test 3 was used.

#### Human experiment

Humans performed the mirror and repeat experiments in a within-participants design (presented in a counterbalanced order across participants). They were first exposed to the grammar during two 64-trial blocks using the same length-4 training sequences as for baboons. They were then presented with Test 3, using the same sequences as used for baboons (i.e. the same 48 length-6 baseline sequences, 16 length-6 consistent and 16 length-6 inconsistent sequences, each presented twice).

### Data analyses

Incorrect trials were removed from the data set (3.35%), as well as RTs greater than three standard deviations from the mean (2.43% of the remaining trials; computed for each participant, target and test, after removal of trials with any abnormally long RT, i.e. > 3 seconds). The dependent variable used in the analyses was the response times on the different targets (i.e., the time elapsed between the appearance of each target and the participant’s touch). Two-tailed paired t-tests with Bonferroni–Holm correction were used to assess the difference in RTs between consistent and inconsistent sequences for each experiment (Mirror, Repeat, i.e. n = 2) and target (Target 4, Target 5, Target 6, i.e. n = 3) for both species: adjusted alpha level = 0.05/(6 – rank number of pair by degree of significance +1)), as well as for baboons within each test (Test 1, Test 2, Test 3, i.e. n = 3) of the mirror experiment for Target 5: adjusted alpha level = 0.05/(3 – rank number of pair by degree of significance +1). Two-tailed t-tests with Bonferroni–Holm correction were used to assess the difference in RTs between baboons and humans for each target (Target 4, Target 5, Target 6, i.e. n = 3) and condition (Consistent, Inconsistent, i.e. n = 2), in Test 3 of the mirror experiment: adjusted alpha level = 0.05/(6 – rank number of pair by degree of significance +1)). Statistical analyses were carried out using R (V3.1.1, www.R-project.org).

## Supplementary information

Supplementary Information.

## Data Availability

The datasets analysed during the current study are available in the figshare repository, https://figshare.com/s/d9fe2b80ef4bed8068cd.
