# Supplementary material for: Baboons (Papio papio) Process a Context-Free but Not a Context-Sensitive Grammar
Source: Sci Rep. 2020 Apr 30;10:7381. doi: 10.1038/s41598-020-64244-5 (PMC7193559; doi:10.1038/s41598-020-64244-5)
Supplement: Supplementary file 1 — Supplementary Information. [file 41598_2020_64244_MOESM1_ESM.pdf]

Supplementary Information:

Baboons (*Papio papio*) Process a Context-Free but Not a Context-Sensitive Grammar

Raphaelle Malassis (1, 2), Stanislas Dehaene (3, 4) & Joël Fagot (1)

1 – Laboratoire de Psychologie Cognitive, Université d'Aix-Marseille, Marseille, France.

2 – School of Psychology and Neuroscience, University of St Andrews, St Andrews, Fife, Scotland, United Kingdom.

3 – Collège de France, Paris, France.

4 – Cognitive Neuroimaging Unit, CEA DSV/I2BM, INSERM, Université Paris Sud, Université Paris-Saclay, NeuroSpin Center, 91191 Gif-sur-Yvette, France.

Corresponding author: raphaelle.malassis@gmail.com

Supplementary Figure 1. Locations on the touchscreen. Letters are given for illustrative purposes but were not displayed.

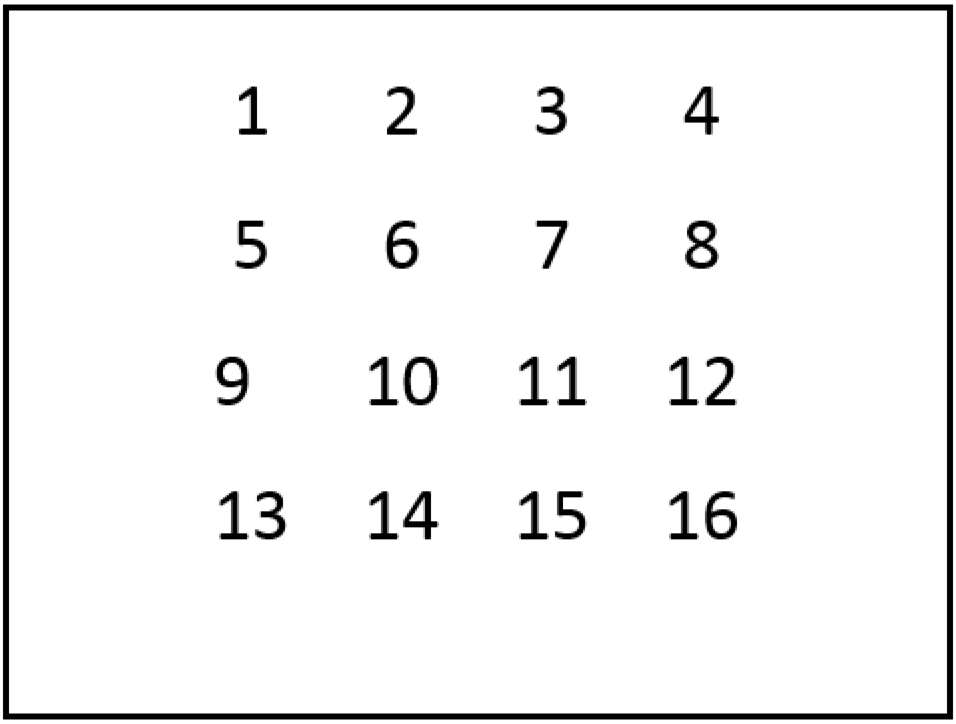

Supplementary Table 1. Mean response times and standard deviation (SD)

Population	Grammar	Test	Target	Condition	N	Mean (ms)	SD (ms)
Baboons	Mirror	1	4	Consistent	14	343	79
Baboons	Mirror	1	4	Inconsistent	14	347	81
Baboons	Mirror	1	5	Consistent	14	415	42
Baboons	Mirror	1	5	Inconsistent	14	429	43
Baboons	Mirror	1	6	Consistent	14	497	91
Baboons	Mirror	1	6	Inconsistent	14	482	101
Baboons	Mirror	2	4	Consistent	14	376	61
Baboons	Mirror	2	4	Inconsistent	14	380	61
Baboons	Mirror	2	5	Consistent	14	410	37
Baboons	Mirror	2	5	Inconsistent	14	433	33
Baboons	Mirror	2	6	Consistent	14	500	92
Baboons	Mirror	2	6	Inconsistent	14	493	76
Baboons	Mirror	3	4	Consistent	14	382	70
Baboons	Mirror	3	4	Inconsistent	14	385	65
Baboons	Mirror	3	5	Consistent	14	437	50
Baboons	Mirror	3	5	Inconsistent	14	457	46
Baboons	Mirror	3	6	Consistent	14	525	87
Baboons	Mirror	3	6	Inconsistent	14	521	92
Baboons	Repeat	1	4	Consistent	14	444	54
Baboons	Repeat	1	4	Inconsistent	14	440	45
Baboons	Repeat	1	5	Consistent	14	425	48
Baboons	Repeat	1	5	Inconsistent	14	414	44
Baboons	Repeat	1	6	Consistent	14	473	59
Baboons	Repeat	1	6	Inconsistent	14	473	68
Baboons	Repeat	2	4	Consistent	14	471	50
Baboons	Repeat	2	4	Inconsistent	14	475	52
Baboons	Repeat	2	5	Consistent	14	431	36
Baboons	Repeat	2	5	Inconsistent	14	420	46
Baboons	Repeat	2	6	Consistent	14	486	89
Baboons	Repeat	2	6	Inconsistent	14	495	77
Baboons	Repeat	3	4	Consistent	14	478	48
Baboons	Repeat	3	4	Inconsistent	14	489	51
Baboons	Repeat	3	5	Consistent	14	452	48
Baboons	Repeat	3	5	Inconsistent	14	448	49
Baboons	Repeat	3	6	Consistent	14	512	69
Baboons	Repeat	3	6	Inconsistent	14	505	69
Humans	Mirror	3	4	Consistent	8	462	80
Humans	Mirror	3	4	Inconsistent	8	453	65
Humans	Mirror	3	5	Consistent	8	450	70
Humans	Mirror	3	5	Inconsistent	8	607	74
Humans	Mirror	3	6	Consistent	8	436	77
Humans	Mirror	3	6	Inconsistent	8	422	67
Humans	Repeat	3	4	Consistent	8	447	82
Humans	Repeat	3	4	Inconsistent	8	437	104
Humans	Repeat	3	5	Consistent	8	472	83
Humans	Repeat	3	5	Inconsistent	8	578	90
Humans	Repeat	3	6	Consistent	8	417	68
Humans	Repeat	3	6	Inconsistent	8	413	97

Supplementary Table 2. Sets of sequences used in the different phases. Numbers refer to locations on the touchscreen**.**

Set	Mirror	Repeat
4-target training sequences (Exposure phases)	4.1.1.4	1.4.1.4
	11.1.1.11	1.11.1.11
	12.1.1.12	1.12.1.12
	15.1.1.15	1.15.1.15
	1.2.2.1	2.1.2.1
	7.2.2.7	2.7.2.7
	8.2.2.8	2.8.2.8
	9.2.2.9	2.9.2.9
	1.3.3.1	3.1.3.1
	4.3.3.4	3.4.3.4
	5.3.3.5	3.5.3.5
	11.3.3.11	3.11.3.11
	6.4.4.6	4.6.4.6
	11.4.4.11	4.11.4.11
	14.4.4.14	4.14.4.14
	16.4.4.16	4.16.4.16
	4.5.5.4	5.4.5.4
	10.5.5.10	5.10.5.10
	13.5.5.13	5.13.5.13
	16.5.5.16	5.16.5.16
	1.6.6.1	6.1.6.1
	3.6.6.3	6.3.6.3
	11.6.6.11	6.11.6.11
	16.6.6.16	6.16.6.16
	5.7.7.5	7.5.7.5
	6.7.7.6	7.6.7.6
	10.7.7.10	7.10.7.10
	14.7.7.14	7.14.7.14
	5.8.8.5	8.5.8.5
	9.8.8.9	8.9.8.9
	12.8.8.12	8.12.8.12
	15.8.8.15	8.15.8.15
	4.9.9.4	9.4.9.4
	7.9.9.7	9.7.9.7
	12.9.9.12	9.12.9.12
	13.9.9.13	9.13.9.13
	1.10.10.1	10.1.10.1
	8.10.10.8	10.8.10.8
	12.10.10.12	10.12.10.12
	14.10.10.14	10.14.10.14
	5.11.11.5	11.5.11.5
	10.11.11.10	11.10.11.10
	14.11.11.14	11.14.11.14
	16.11.11.16	11.16.11.16
	2.12.12.2	12.2.12.2
	3.12.12.3	12.3.12.3
	6.12.12.6	12.6.12.6
	13.12.12.13	12.13.12.13
	2.13.13.2	13.2.13.2
	3.13.13.3	13.3.13.3
	8.13.13.8	13.8.13.8
	10.13.13.10	13.10.13.10
	2.14.14.2	14.2.14.2
	8.14.14.8	14.8.14.8
	9.14.14.9	14.9.14.9
	15.14.14.15	14.15.14.15
	2.15.15.2	15.2.15.2
	6.15.15.6	15.6.15.6
	7.15.15.7	15.7.15.7
	13.15.15.13	15.13.15.13
	3.16.16.3	16.3.16.3
	7.16.16.7	16.7.16.7
	9.16.16.9	16.9.16.9
	15.16.16.15	16.15.16.15

Set	Mirror	Repeat
6-target baseline sequences (Test 1)	15.2.1.1.2.15	1.2.15.1.2.15
	13.10.2.2.10.13	2.10.13.2.10.13
	1.15.3.3.15.1	3.15.1.3.15.1
	3.1.10.10.1.3	10.1.3.10.1.3
	10.3.13.13.3.10	13.3.10.13.3.10
	2.13.15.15.13.2	15.13.2.15.13.2
6-target baseline sequences (Tests 2-3)	11.3.1.1.3.11	1.3.11.1.3.11
	6.4.1.1.4.6	1.4.6.1.4.6
	4.6.1.1.6.4	1.6.4.1.6.4
	12.10.1.1.10.12	1.10.12.1.10.12
	3.11.1.1.11.3	1.11.3.1.11.3
	10.12.1.1.12.10	1.12.10.1.12.10
	12.8.2.2.8.12	2.8.12.2.8.12
	8.12.2.2.12.8	2.12.8.2.12.8
	11.1.3.3.1.11	3.1.11.3.1.11
	1.11.3.3.11.1	3.11.1.3.11.1
	6.1.4.4.1.6	4.1.6.4.1.6
	1.6.4.4.6.1	4.6.1.4.6.1
	14.9.4.4.9.14	4.9.14.4.9.14
	16.11.4.4.11.16	4.11.16.4.11.16
	9.14.4.4.14.9	4.14.9.4.14.9
	11.16.4.4.16.11	4.16.11.4.16.11
	16.7.5.5.7.16	5.7.16.5.7.16
	7.16.5.5.16.7	5.16.7.5.16.7
	4.1.6.6.1.4	6.1.4.6.1.4
	1.4.6.6.4.1	6.4.1.6.4.1
	16.5.7.7.5.16	7.5.16.7.5.16
	5.16.7.7.16.5	7.16.5.7.16.5
	12.2.8.8.2.12	8.2.12.8.2.12
	2.12.8.8.12.2	8.12.2.8.12.2
	15.13.8.8.13.15	8.13.15.8.13.15
	13.15.8.8.15.13	8.15.13.8.15.13
	14.4.9.9.4.14	9.4.14.9.4.14
	4.14.9.9.14.4	9.14.4.9.14.4
	12.1.10.10.1.12	10.1.12.10.1.12
	1.12.10.10.12.1	10.12.1.10.12.1
	3.1.11.11.1.3	11.1.3.11.1.3
	1.3.11.11.3.1	11.3.1.11.3.1
	16.4.11.11.4.16	11.4.16.11.4.16
	4.16.11.11.16.4	11.16.4.11.16.4
	10.1.12.12.1.10	12.1.10.12.1.10
	8.2.12.12.2.8	12.2.8.12.2.8
	2.8.12.12.8.2	12.8.2.12.8.2
	1.10.12.12.10.1	12.10.1.12.10.1
	15.8.13.13.8.15	13.8.15.13.8.15
	8.15.13.13.15.8	13.15.8.13.15.8
	9.4.14.14.4.9	14.4.9.14.4.9
	4.9.14.14.9.4	14.9.4.14.9.4
	13.8.15.15.8.13	15.8.13.15.8.13
	8.13.15.15.13.8	15.13.8.15.13.8
	11.4.16.16.4.11	16.4.11.16.4.11
	7.5.16.16.5.7	16.5.7.16.5.7
	5.7.16.16.7.5	16.7.5.16.7.5
	4.11.16.16.11.4	16.11.4.16.11.4

Set	Mirror		Repeat	
	Consistent	Inconsistent	Consistent	Inconsistent
Test 1	11.14.5.5.14.11	11.14.5.5.11.14	11.12.8.11.12.8	11.12.8.11.8.12
	11.7.6.6.7.11	11.7.6.6.11.7	11.8.12.11.8.12	11.8.12.11.12.8
	12.16.14.14.16.12	12.16.14.14.12.16	12.5.6.12.5.6	12.5.6.12.6.5
	12.8.11.11.8.12	12.8.11.11.12.8	12.6.5.12.6.5	12.6.5.12.5.6
	14.11.5.5.11.14	14.11.5.5.14.11	14.12.16.14.12.16	14.12.16.14.16.12
	14.7.9.9.7.14	14.7.9.9.14.7	14.16.12.14.16.12	14.16.12.14.12.16
	16.12.14.14.12.16	16.12.14.14.16.12	16.4.9.16.4.9	16.4.9.16.9.4
	16.5.8.8.5.16	16.5.8.8.16.5	16.9.4.16.9.4	16.9.4.16.4.9
	4.9.16.16.9.4	4.9.16.16.4.9	4.6.8.4.6.8	4.6.8.4.8.6
	4.9.7.7.9.4	4.9.7.7.4.9	4.8.6.4.8.6	4.8.6.4.6.8
	5.16.8.8.16.5	5.16.8.8.5.16	5.11.14.5.11.14	5.11.14.5.14.11
	5.6.12.12.6.5	5.6.12.12.5.6	5.14.11.5.14.11	5.14.11.5.11.14
	6.5.12.12.5.6	6.5.12.12.6.5	6.11.7.6.11.7	6.11.7.6.7.11
	6.8.4.4.8.6	6.8.4.4.6.8	6.7.11.6.7.11	6.7.11.6.11.7
	7.11.6.6.11.7	7.11.6.6.7.11	7.4.9.7.4.9	7.4.9.7.9.4
	7.14.9.9.14.7	7.14.9.9.7.14	7.9.4.7.9.4	7.9.4.7.4.9
	8.12.11.11.12.8	8.12.11.11.8.12	8.16.5.8.16.5	8.16.5.8.5.16
	8.6.4.4.6.8	8.6.4.4.8.6	8.5.16.8.5.16	8.5.16.8.16.5
	9.4.16.16.4.9	9.4.16.16.9.4	9.14.7.9.14.7	9.14.7.9.7.14
	9.4.7.7.4.9	9.4.7.7.9.4	9.7.14.9.7.14	9.7.14.9.14.7

	Mirror		Repeat	
Set	Consistent	Inconsistent	Consistent	Inconsistent
Test 2	10.13.5.5.13.10	10.13.5.5.10.13	10.14.8.10.14.8	10.14.8.10.8.14
	13.10.5.5.10.13	13.10.5.5.13.10	10.8.14.10.8.14	10.8.14.10.14.8
	13.3.12.12.3.13	13.3.12.12.13.3	12.13.3.12.13.3	12.13.3.12.3.13
	14.8.10.10.8.14	14.8.10.10.14.8	12.3.13.12.3.13	12.3.13.12.13.3
	15.2.14.14.2.15	15.2.14.14.15.2	14.15.2.14.15.2	14.15.2.14.2.15
	16.3.6.6.3.16	16.3.6.6.16.3	14.2.15.14.2.15	14.2.15.14.15.2
	2.15.14.14.15.2	2.15.14.14.2.15	15.6.7.15.6.7	15.6.7.15.7.6
	3.13.12.12.13.3	3.13.12.12.3.13	15.7.6.15.7.6	15.7.6.15.6.7
	3.16.6.6.16.3	3.16.6.6.3.16	2.7.9.2.7.9	2.7.9.2.9.7
	4.5.3.3.5.4	4.5.3.3.4.5	2.9.7.2.9.7	2.9.7.2.7.9
	5.4.3.3.4.5	5.4.3.3.5.4	3.4.5.3.4.5	3.4.5.3.5.4
	6.7.15.15.7.6	6.7.15.15.6.7	3.5.4.3.5.4	3.5.4.3.4.5
	7.6.15.15.6.7	7.6.15.15.7.6	5.10.13.5.10.13	5.10.13.5.13.10
	7.9.2.2.9.7	7.9.2.2.7.9	5.13.10.5.13.10	5.13.10.5.10.13
	8.14.10.10.14.8	8.14.10.10.8.14	6.16.3.6.16.3	6.16.3.6.3.16
	9.7.2.2.7.9	9.7.2.2.9.7	6.3.16.6.3.16	6.3.16.6.16.3
Test 3 & Control	1.13.7.7.13.1	1.13.7.7.1.13	1.5.9.1.5.9	1.5.9.1.9.5
	1.16.8.8.16.1	1.16.8.8.1.16	1.9.5.1.9.5	1.9.5.1.5.9
	10.4.15.15.4.10	10.4.15.15.10.4	10.16.2.10.16.2	10.16.2.10.2.16
	12.15.5.5.15.12	12.15.5.5.12.15	10.2.16.10.2.16	10.2.16.10.16.2
	13.1.7.7.1.13	13.1.7.7.13.1	10.3.9.10.3.9	10.3.9.10.9.3
	15.12.5.5.12.15	15.12.5.5.15.12	10.9.3.10.9.3	10.9.3.10.3.9
	15.9.11.11.9.15	15.9.11.11.15.9	11.15.9.11.15.9	11.15.9.11.9.15
	16.1.8.8.1.16	16.1.8.8.16.1	11.9.15.11.9.15	11.9.15.11.15.9
	16.2.10.10.2.16	16.2.10.10.16.2	15.10.4.15.10.4	15.10.4.15.4.10
	2.16.10.10.16.2	2.16.10.10.2.16	15.4.10.15.4.10	15.4.10.15.10.4
	3.9.10.10.9.3	3.9.10.10.3.9	5.12.15.5.12.15	5.12.15.5.15.12
	4.10.15.15.10.4	4.10.15.15.4.10	5.15.12.5.15.12	5.15.12.5.12.15
	5.9.1.1.9.5	5.9.1.1.5.9	7.1.13.7.1.13	7.1.13.7.13.1
	9.15.11.11.15.9	9.15.11.11.9.15	7.13.1.7.13.1	7.13.1.7.1.13
	9.3.10.10.3.9	9.3.10.10.9.3	8.1.16.8.1.16	8.1.16.8.16.1
	9.5.1.1.5.9	9.5.1.1.9.5	8.16.1.8.16.1	8.16.1.8.1.16
